# Validation of Nonlinear Dependence of Rolling Friction Moment on the Normal Force for Elastic Materials

**DOI:** 10.3390/ma15072518

**Published:** 2022-03-29

**Authors:** Stelian Alaci, Florina-Carmen Ciornei, Ionut-Cristian Romanu

**Affiliations:** Mechanics and Technologies Department, Stefan cel Mare University of Suceava, 720229 Suceava, Romania; florina.ciornei@usm.ro (F.-C.C.); ionutromanucristian@usm.ro (I.-C.R.)

**Keywords:** elastic materials, nonlinear rolling friction model, inclined plane

## Abstract

Analogous to the Amonton–Coulomb relation, which states the linear dependency between the dynamic sliding friction and the normal reaction, the rolling friction moment is commonly accepted as proportional to the normal reaction in a concentrated point contact. This hypothesis persists since it gives simple dynamic models and also due to difficulties met in experimental estimations of the rolling friction torques. Recent theoretical studies proved that this dependency is nonlinear even for elastic materials. A special rotor is designed, with an adjustable position for the center of mass but with constant mass and constant axial inertia moment. The pure rolling motion of the rotor on an inclined controlled small slope is studied. The angular acceleration of motion is theoretically deduced, assuming that the rolling friction torque is proportional to the normal force raised at a certain power. The deduced theoretical dynamic model evidences the influence of the eccentricity of the rotor upon the acceleration. For the particular case of linear dependency—the exponent of the power equal to one, the law of motion is independent of the configuration of the rotor. Experimental tests were made using the rotor constructed according to the theoretical model. For two positions of the center of mass, the experimental law of motion on the inclined plane is established by a non-contact method and the two different laws obtained to validate the nonlinear dependence rolling friction torque-normal force. The paper validates in an experimental manner the considered nonlinear assumption. The experimental tests concerning the microtopography of the contacting surfaces reveal that the hypothesis required by Hertzian theory, namely smooth contacting surfaces, is not satisfied. Thus, the distribution of pressure on the contact area does not obey the Hertzian semi-ellipsoidal distribution and further experimental tests are required for quantitative findings on the rolling friction torque-normal force relationship.

## 1. Introduction

### 1.1. General Context and Purpose of the Work

Two bodies can interact by means of field or by direct contact. For solid bodies, two main situations are distinguished: conform and nonconform contact [1]. In the case of nonconform contacts, the relative velocity of the theoretical contact points decides the type of motion [2]. The case of pure rolling, characteristic of nil relative velocity, is the aim of numerous technical applications due to reduced power losses and diminished wear [3,4,5]. The first example is the wheel, from the early ancient shapes to the modern uses and designs. Rolling bearings are most notable and widespread; their importance is confirmed by the numerous comprehensive theoretical and experimental studies [6,7,8,9].

Rolling friction is a subject related to the losses from rolling contacts, and it has been studied by classical methods and dedicated equipment [10,11,12,13]. The modern and various engineering applications of rolling motion such as spherical robots [14], granular materials from pharmacy [15], or as solid lubricants [16] in agriculture [17], sports [18], and mainly automotive [19] require new investigation methods and techniques. The methods based on oscillatory motion can be exemplified [20,21,22,23] and also the techniques founded on non-oscillatory motions. From the last category, the inclined plane method in the study of rolling friction is well known [24,25] and an example of the method is proposed in the current work. 

### 1.2. The Friction Torsor

**Figure 1:**
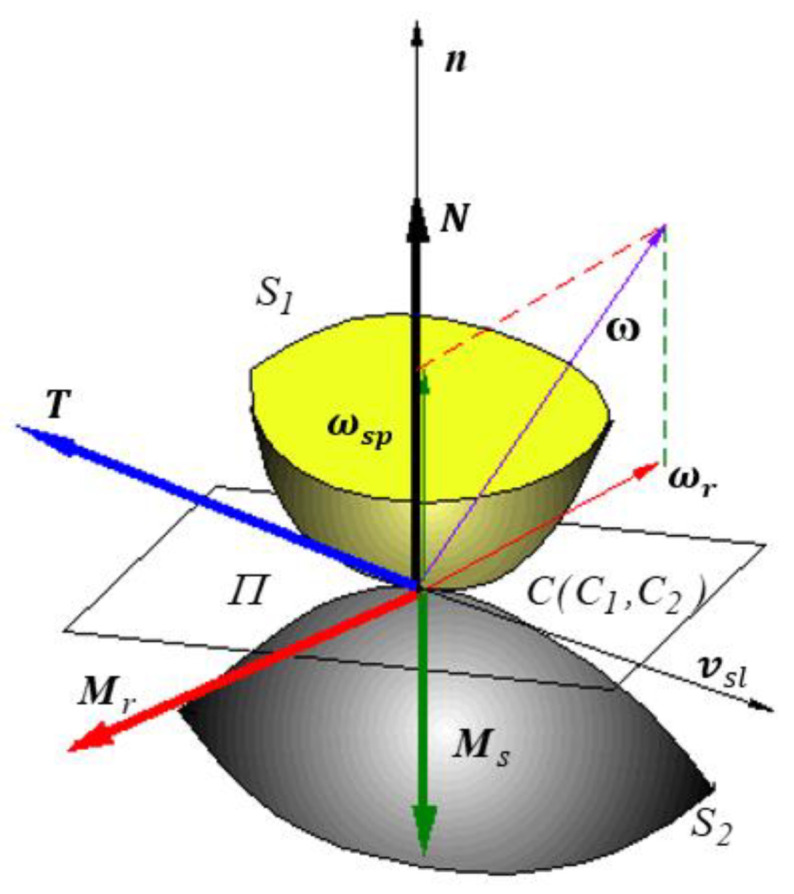
The components of friction torsor in a Hertzian point contact.

The contacts between solid elastic bodies can be conforming or nonconforming (Hertzian). In the case of Hertzian contact, the theoretical dimension of the contact area is zero, no matter if the contact is a point or line. The contact area for the Hertzian point contact is of elliptic shape and the normal pressure presents a semi-ellipsoidal distribution [26,27]. For the Hertzian point contact (Figure 1), the relative motion between the two bodies is described by the relative sliding velocity $v_{sl}$ between the two points that have coincident positions and the angular relative velocity *ω*. Unlike the sliding velocity that is contained in the common tangent plane (Π) in the contact point *C*, the angular relative velocity has an arbitrary orientation, with a spinning component $\omega_{sp}$ along the normal in the contact point and a rolling component $\omega_{r}$ contained in the tangent plane. To these three kinematics vectors, the following components of the friction torsor in the contact point correspond [28]:

- the sliding friction force ***T*** of the opposite direction of sliding velocity $v_{sl}$;
- the spinning friction torque $M_{s}$, parallel and of the opposite direction of the spinning angular velocity $\omega_{sp}$;
- the rolling friction torque, $M_{r}$, parallel and of the opposite direction of the rolling angular velocity $\omega_{r}$.

For the unlubricated contacts, the friction force is modeled by the Amonton–Coulomb law, being proportional to the normal force when the velocity is non-zero, the proportionality factor being the coefficient of dynamic friction $\mu_{d}$. When the sliding velocity is zero, the friction force is described by the static friction coefficient, $\mu_{s}$ representing the ratio between the maximum static friction force and the normal reaction. To be mentioned that the value of the coefficient of static friction is greater than the coefficient of dynamic friction. 

The spinning friction torque is estimated based on the hypothesis that the ellipsoidal pressure distribution on the contact area generates elementary friction forces on elementary contact surfaces. The spinning torque, calculated as the results of all moments generated by elementary friction forces, is proportional to the normal force raised at power 4/3 [29]. 

A current subject is the dependency of the rolling friction torque on the normal force. In mechanics monographs, it is accepted as the first hypothesis, by analogy to the sliding friction law of Amonton–Coulomb, that a proportionality exists between the rolling friction moment and the normal force, and the proportionality factor is the coefficient of rolling friction $s_{r}$, with dimension of length [28,30]. To be mentioned that the rolling friction can only be explained if the hypothesis of rigid bodies is dropped and the deformable bodies are considered in contact. In this case, for the Herzian point contact of two bodies of revolution, the normal reaction is eccentric with respect to the plane made by the rotation axis of the two bodies. Based on the theory of elasticity, Cherepanov [31] reaches the conclusion that the moment of rolling friction is, like the spinning friction moment, proportional to the normal force raised at power 4/3. The comprehension of this dependency is of actuality for the automotive industry and rolling stock in the rail transport industry, where the rolling friction moment permits the estimation of the actual lost work. 

There are two methods of estimation of the moment of rolling friction. The first method involves oscillatory motion between the two bodies; pendula devices are used and the damping of motion is used for determining the rolling friction torque. The method presents the advantage that the region where the contact point between the pendulum and frame is reduced, and thus, the variation of mechanical properties is eliminated. The main drawback of the method is the advanced mathematical apparatus required by the nonlinear differential equations describing the oscillations of the pendula. The hypothesis of nonlinear dependency between the rolling friction torque and the normal force leads to an increased degree of nonlinearity of the differential equation of motion, making inappropriate the classical numerical integration methods (like Runge-Kutta [32]) and requiring special methods of numerical integration. The second method concerns non-oscillatory motions and is to study the motion of a body with rotational symmetry along an inclined plane.

### 1.3. Models of Rolling Friction Torque

A qualitative explanation of the rolling friction dependency could be the fact that the moment of rolling friction increases with the increasing deformations of the contacting bodies. It is experimentally revealed that rolling friction torque increases as long as the deformations enlarge. For example, for two balls of the same size, a steel one and a rubber one, resting on the same plate, the rolling friction moments will differ, having significantly smaller value for the steel ball [33,34]. It is known, from the theory of Hertzian contact, that the deformations of the contacting bodies are dependent on the elastic characteristics of the two bodies: Young modulus *E* and Poisson coefficient ν, [27]. 

**Figure 2:**
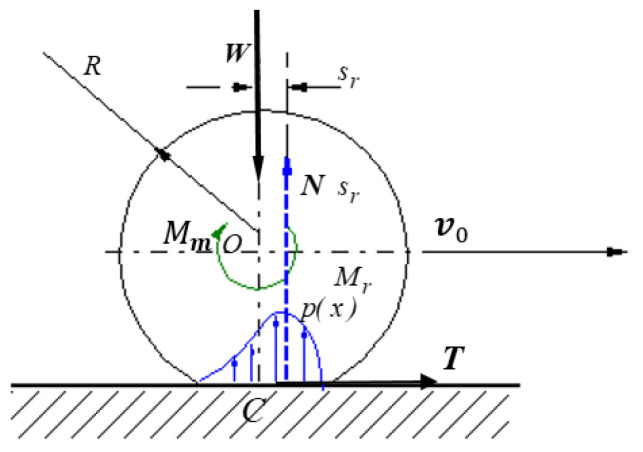
Eccentric position of the normal reaction in a rolling Hertzian contact.

In Figure 2, the normal load W  generates on the contact area around the initial contact point a symmetrical pressure distribution p(x) having the resultant a force N passing through the center of the body *O*. When a motor torque $M_{m}$ is applied, the body starts to move with the velocity $v_{0}$ and the pressure distribution becomes asymmetric, and thus, the resultant N will be placed at a distance $s_{r}$ towards the direction of motion. 

The proportionality between the modulus of the rolling friction torque and the magnitude of the normal force is the simplest hypothesis that expresses, as Cherepanov shows, the Coulomb law for rolling friction:

(1) $$M_{r}=TR=kN$$

where $M_{r}$ is the rolling friction moment, T the friction force, N the normal reaction and *R* is the rolling radius. The Equation (1) is obtained by similitude to the Amonton-Coulomb law for the sliding friction force and normal reaction dependence:

(2) T=μN

Recently, based on elastic contact theory considerations, Cherepanov [31] demonstrates that the coefficient *k* from Equation (1) is not a constant but depends on the normal pressing force N obeying the laws (3) for cylindrical rolling bodies like wheels, where N is the force per unit length of the cylinder and (4) for spherical bodies:

(3) $$k=\eta_{w}{\left(NRP\right)}^{1/2}$$

(4) $$k=\eta_{b}{\left(NRP\right)}^{1/3}$$

Thus, for bodies of revolution, $\eta_{w},\;\eta_{b},$ are the dimensionless rolling coefficients and *P* is the elastic compliance of the two contacting bodies:

(5) $$P=\frac{1-\nu_{1}^{2}}{E_{1}}+\frac{1-\nu_{2}^{2}}{E_{2}}$$

where $E_{1,2};\;\nu_{1,2}$ are the Young moduli and Poisson ratios, respectively. 

From Equations (3) and (4), the rolling friction moment for cylinders can be expressed as:

(6) $$M_{r}=k_{w}{\left(N\right)}^{3/2}$$

and for spherical rolling bodies:

(7) $$M_{r}=k_{b}{\left(N\right)}^{4/3}$$

The Equations (6) and (7) can be expressed in a general manner for any rolling body:

(8) $$M_{r}=s_{r}N^{\lambda }$$

**Figure 3:**
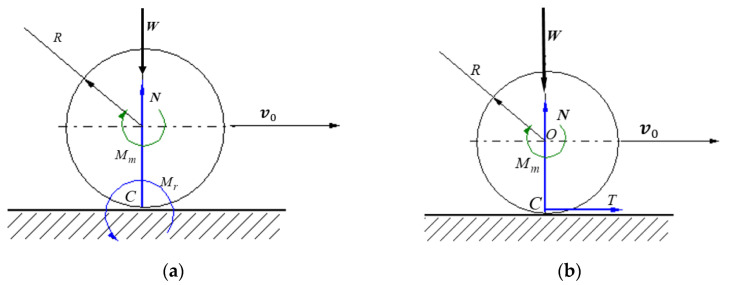
Characterization of rolling friction by means of (**a**) the rolling friction torque; (**b**) the friction force.

The simplest models of rolling friction torque assume λ=1, and a linear dependence between the rolling friction moment and the normal resistance $M_{r}=s_{r}N$ [28,30]. To be noted, in this case, $s_{r}$ has the dimension of length. The eccentric force N can be replaced by a normal force N passing through the center of the body and a moment $M_{r}=s_{r}N$, or by a peripheral force T, $M_{r}=TR$ that acts in the theoretical point of contact *C* (Figure 3). 

The main goal of the paper is to obtain the equation of motion of a rotor and to validate experimentally the nonlinear dependency (Equation (8)) between the rolling friction moment and the normal force.

## 2. Materials and Methods

### 2.1. Proposed Methodology and Theoretical Background

The principle of inclined plane was chosen for the present work, though the main drawback of the method consists in the fact that the effect of rolling friction can be evidenced for very reduced values of the tilting angle smaller than one degree. 

**Figure 4:**
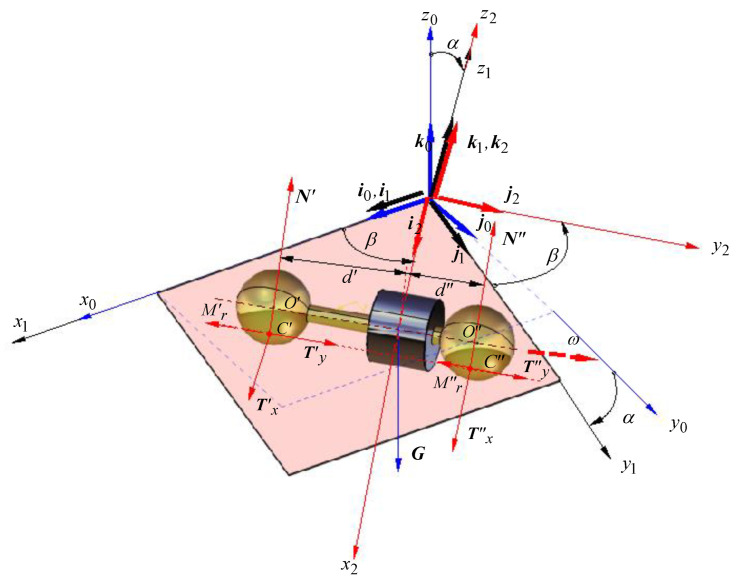
The rotor on the inclined plane and attached coordinate systems.

The rolling body can be any body of rotational symmetry made of homogenous material, and the most convenient is to employ cylinders or spheres. The cylindrical bodies have the disadvantage of making linear contact with the plane, and thus, the contact pressure distribution presents the edge effect [35]. When a complex revolution body made of two cylinders is used, the constraint of coaxial axes must be strictly obeyed. When a spherical body is used, the disadvantages mentioned for the cylindrical bodies are eliminated but the sphere–plane contact introduces the drawback of a greater number of degrees of freedom compared to a cylinder–plane contact. In theory, the motion is a rolling motion about an axis normal to the line of the greatest slope of the plane. In fact, the moment generated by the distribution of elementary forces on the contact area has as components the rolling torque and the spinning torque that makes the sphere rotate about an axis normal to the plane. To overcome this aspect, the work proposes a specially constructed rotor having a unique axis of symmetry of rotation, axis meant to be the kinematical rotation axis at the same time. For this purpose, it was proposed that the rotor is constructed from two identical spheres, connected rigidly by a rod, with the condition the centers of the balls O′ and O″ are precisely set on the axis of the rod, as shown in Figure 4. The rotor obtained makes two point-Hertz type contacts with the inclined plane. The advantage of this rotor consists of the fact that for small values of the angle of inclination of the plane (*α*), the body performs pure rolling and maintains the direction of the axis of rotation. 

**Figure 5:**
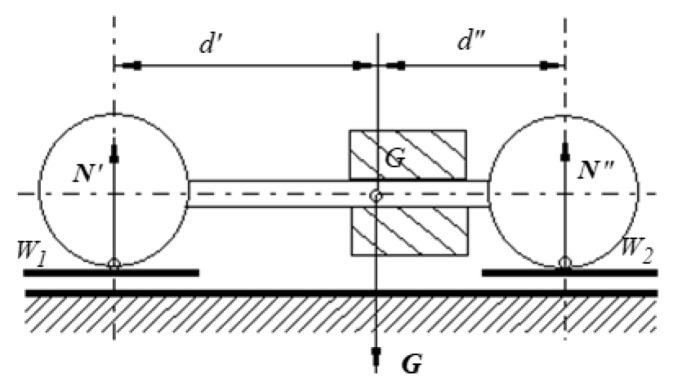
Finding the position of the center of mass *G* from the free body diagram of the rotor.

The motion of the rotor under an angle with respect to the line of greatest slope of the plane (*β*) is equivalent to a rolling along the line of greatest slope for a plane with a much smaller angle of inclination, the angle that can be precisely determined by calculus. A collar is mounted on the coupling rod. It can slide co-axially along the rod, being fixed rod with a hex socket set with a flat point. Thus, by adjusting the position of the collar, the position of the center of mass of the assembly *C* can be modified while the mass and the moment of inertia with respect to the rotation axis remain unchanged. The center of mass of the rotor is found by the next methodology: the balls of the rotor are placed on two weighing scales $W_{1}$, $W_{2}$ and the indications represent the masses which generate the reactions *N*’, *N*”, as presented in the scheme from Figure 5. The rotor can be regarded as a beam on two supports. From the moment equations applied for the free body diagram, the values of *d*′ and *d*″, characterizing the position of *G*, are obtained. The outer diameter of the cylindrical part must be noticeably smaller than the diameter of the spheres because only the balls should be in contact with the inclined plane.

To obtain the inclined plane with the attached frame $x_{2}y_{2}z_{2}$, the $\;x_{0}y_{0}z_{0}$ system of coordinate having the axes $Ox_{0}$ and $Oy_{0}$ placed in a horizontal plane is considered. A rotation of angle (−α) about the $Ox_{0}$ axis is applied and the coordinate system takes the new position $x_{1}y_{1}z_{1}$, with the mention that $Ox_{0}Ox_{1}$. This rotation is described by the matrix [36]:

(9) $$R_{x}\left(\alpha \right)=\left[\begin{array}{ccc} 1 & 0 & 0\\ 0 & \cos\left(-\alpha \right) & -\sin\left(-\alpha \right)\\ 0 & \sin\left(-\alpha \right) & \cos\left(-\alpha \right) \end{array}\right]$$

Next, a rotation of angle β about the $z_{1}$ axis is applied, being described by the matrix [34]:

(10) $$R_{z}\left(\beta \right)=\left[\begin{array}{ccc} \cos\beta & -\sin\beta & 0\\ \sin\beta & \cos\beta & 0\\ 0 & 0 & 1 \end{array}\right]$$

The coordinate system is now in the position $x_{2}y_{2}z_{2}$, $Oz_{1}\equiv Oz_{2}$. In the frame $x_{2}y_{2}z_{2}$, the plane $x_{2}y_{2}$ is the rolling plane and the $Ox_{2}$ axis is the direction of motion of the rotor while the axis $Oy_{2}$ is the rolling direction. 

From the exterior, only the weight force, placed in the center of mass *G* of the rotor, is acting upon the body. In general, when the rotor takes an asymmetrical position—characterized by the distances d′ and d″ from the centers of the balls O′ and O″ to the center of mass of the rotor, it is considered, without diminishing the generality of the problem, that the motion of the rotor is made in a way ensuring that the center of mass is placed in the plane $x_{2}z_{2}$. The only force acting upon the rotor is the gravity G directed on the axis $z_{0}$:

(11) $$G=Mgk_{0}$$

where M is the mass of the rotor and g is the free-fall acceleration. In the points of contact C′ and C″ the reactions from the plane will act, being composed of:

- normal reactions:
  (12) $$N^{\prime }=N^{\prime }k_{2},\;N^{\prime }=N^{\prime }k_{2}$$
- tangential reactions, placed in the $x_{2}y_{2}$ plane. The components on $Ox_{2}$ are:
  (13) $$T_{x}^{\prime }=T_{x}^{\prime }i_{2},\;T_{x}^{\prime \prime }=T_{x}^{\prime \prime }i_{2}$$
  and the projections on the $Oy_{2}$:
  (14) $$T_{y}^{\prime }=T_{y}^{\prime }j_{2},\;T_{y}^{\prime \prime }=T_{y}^{\prime \prime }j_{2}$$
- the rolling torques $M_{r}^{\prime }$ and $M_{r}^{\prime \prime }$ occurring in the contact points, oppose to the rotation *ω* of the rotor about the axis $Oy_{2}$:
  (15) $$M_{r}^{\prime }=-M_{r}^{\prime }j_{2},\;M_{r}^{\prime \prime }=-M_{r}^{\prime \prime }j_{2}$$

In order to find the unknowns of the problem, the linear momentum theorem [37] is applied, for a particular case of the center of mass:

(16) $$Ma_{C}=G+N^{\prime }+N^{\prime \prime }+T_{x}^{\prime }+T_{x}^{\prime \prime }+T_{y}^{\prime }+T_{y}^{\prime \prime }$$

and the angular momentum theorem [35], with respect to the center of mass:

(17) $$J_{C}\varepsilon +\hat{\omega }J_{C}\omega =CC^{\prime }\times \left(N^{\prime }+T_{x}^{\prime }+T_{y}^{\prime }\right)+CC^{\prime \prime }\times \left(N^{\prime }+T_{x}^{\prime }+T_{y}^{\prime }\right)+M_{r}^{\prime }+M_{r}^{\prime \prime }$$

where $a_{C}$ is the acceleration of the center of mass:

(18) $$a_{C}=\frac{d^{2}}{dt^{2}}\left(x_{2C}i_{2}+rk_{2}\right)=\frac{d^{2}x_{2C}}{dt^{2}}i_{2}={\ddot{x}}_{2C}i_{2}$$

and $CC^{\prime }$ and $CC^{\prime \prime }$ are the position vectors of the points of contact with respect to the center of mass:

(19) $$CC^{\prime }=-d^{\prime }j_{2}-rk_{2},\;CC^{\prime \prime }=d^{\prime \prime }j_{2}-rk_{2}$$

In Equation (17) $J_{C}$ is the matrix of the moments of inertia [37] with respect to a frame with the origin in the center of mass and with axes parallel to the axes of the $x_{2}y_{2}z_{2}$ coordinate system:

(20) $$J_{C}=\left[\begin{array}{ccc} J_{Cx} & 0 & 0\\ 0 & J_{Cy} & 0\\ 0 & 0 & J_{Cz} \end{array}\right]$$

The angular acceleration vector is *ε*:

(21) $$\varepsilon =\left[\begin{array}{c} 0\\ \varepsilon_{y}\\ 0 \end{array}\right]$$

and

(22) $$\hat{\omega }=\left[\begin{array}{ccc} 0 & 0 & \omega \\ 0 & 0 & 0\\ -\omega & 0 & 0 \end{array}\right]$$

is the antisymmetric matrix [38] attached to the angular velocity vector $\omega =\omega j_{2}.$ After calculating the left member of Equation (17), it results in a matrix corresponding to the vector $J_{Cy}\varepsilon j_{2}$. 

From Equation (16) of the impulse-momentum theorem, required for finding the motion and the reactions, all vectors are expressed using the projections on the axes of the system $x_{2}y_{2}z_{2}$ with the exception of the gravity *G*, which is expressed by the projections on the axes of the $x_{0}y_{0}z_{0}$ frame. Let’s express all the vectors from Equation (16) by their projections on the axes of the same frame, and the most convenient is to express the versors of the system $x_{0}y_{0}z_{0}$ as functions of the versors of the system $x_{2}y_{2}z_{2}$. The coordinate system $x_{0}y_{0}z_{0}$ is rotated to overly the system $x_{2}y_{2}z_{2}$ and this displacement denoted $T_{0-2}$ is obtained using the matrix equation [39]:

(23) $$T_{0-2}=R_{x}\left(-\alpha \right)R_{z}\left(\beta \right)$$

The inverse displacement of the coordinate system $x_{2}y_{2}z_{2}$ over the system $x_{0}y_{0}z_{0}$ is obtained with the matrix equation [39]:

(24) $$T_{2-0}={\left[T_{0-2}\right]}^{-1}$$

The result of the calculation is:

(25) $$T_{2-0}=\left[\begin{array}{ccc} \cos\beta & \cos\alpha \sin\beta & -\sin\alpha \sin\beta \\ -\sin\beta & \cos\alpha & -\sin\alpha \cos\beta \\ 0 & \sin\alpha \; & \cos\alpha \end{array}\right]$$

and from here, the final result:

(26) $$k_{0}=-\sin\alpha \sin\beta i_{2}-\sin\alpha \cos\beta j_{2}+\cos\alpha k_{2}$$

Equation (26) is used to express the gravity *G* in the coordinate system $x_{2}y_{2}z_{2}$. Now, the linear momentum theorem (16) conducts to the following three scalar equations expressed by the projections on the axes of the coordinate system $x_{2}y_{2}z_{2}$:

(27) $$M{\ddot{x}}_{2}=T_{x}^{\prime }+T_{x}^{\prime \prime }+Mg\sin\alpha \sin\beta$$

(28) $$0=T_{y}^{\prime }+T_{y}^{\prime \prime }+Mg\sin\alpha \cos\beta$$

(29) $$0=N^{\prime }+N^{\prime \prime }-Mg\cos\alpha$$

The angular momentum theorem, Equation (17), conducts to the next three scalar equations of projection on the axes of the system $x_{2}y_{2}z_{2}:$

(30) $$N^{\prime }d^{\prime }-N^{\prime \prime }d^{\prime \prime }-rT_{y}^{\prime }-rT_{y}^{\prime \prime }=0$$

(31) $$J_{Cy}\varepsilon =-rT_{x}^{\prime }-rT_{x}^{\prime \prime }-M_{r}-M_{t}^{\prime \prime }$$

(32) $$-d^{\prime }T_{x}^{\prime }+d^{\prime \prime }T_{x}^{\prime \prime }=0$$

The unknowns from the system of Equations (27)–(32) are:

- the kinematics parameters *ε* and $a_{C}={\ddot{x}}_{2}$;
- the magnitudes of the reactions from the two points of contact $N^{\prime },\;N^{\prime \prime },\;T_{x}^{\prime },\;T_{x}^{\prime \prime },\;T_{y}^{\prime },\;T_{y}^{\prime \prime },\;M_{r}^{\prime },\;M_{r}^{\prime \prime }$.

At this point, the system has two degrees of freedom, that is the displacement of the center of mass and the rotation about the axis $y_{2}.$ If pure rolling between the spheres and the plane in the contact points is assumed, then a relation between the two motions must exist. The pure rolling condition imposes that the relative velocity from the two pairs sphere-plane should be zero. Since the velocity of any point from the plane is zero, the condition of pure rolling is thus expressed by the relation:

(33) $$v_{C\prime }=v_{C\prime \prime }=0$$

For example, the absolute velocity of the point $C^{\prime }$ is:

(34) $$v_{C\prime }=v_{C}+\omega \times CC^{\prime }={\dot{x}}_{2}i_{2}+\left|\begin{array}{ccc} i_{2} & j_{2} & k_{2}\\ 0 & \omega & 0\\ 0 & -d^{\prime } & -r \end{array}\right|=\left({\dot{x}}_{2}-\omega r\right)i_{2}$$

The condition of pure rolling in the contact point requires that:

(35) $${\dot{x}}_{2}=\omega r$$

One can rapidly verify that Equation (35) ensures pure rolling conditions for the second point of contact *C*”. The time derivative of Equation (35) is:

(36) $${\ddot{x}}_{2}=\varepsilon r$$

The rolling friction torques are assumed as depending on the normal reaction raised at a power:

(37) $$M_{r}^{\prime }=s_{r}N^{\prime \lambda },\;M_{r}^{\prime \prime }=s_{r}N^{\prime \prime \lambda }$$

The system of eight Equations (27)–(32), (35) and (36) is considered and assuming the tribological parameters *s_r_* and λ as known, one can notice that the Equations (27)–(29) can be studied separately, as forming a self-determining system with the unknowns $N^{\prime },\;N^{\prime \prime },\;T_{y}^{\prime },\;T_{y}^{\prime \prime }$. The unknowns $T_{y}^{\prime }$ and $T_{y}^{\prime \prime }$ occur only in the Equations (27) and (29) where they have proportional coefficients and therefore, this fact permits considering a single variable:

(38) $$T_{y}=T_{y}^{\prime }+T_{y}^{\prime \prime }$$

and the system is now a compatible determined one:

(39) $$\{\begin{array}{c} T_{y}+Mg\sin\alpha \cos\beta =0\\ N^{\prime }+N^{\prime \prime }-Mg\cos\alpha =0\\ N^{\prime }d^{\prime }-N^{\prime \prime }d^{\prime \prime }-rT_{y}=0 \end{array}$$

having the solution:

(40) $$T_{y}=-Mg\sin\alpha \cos\beta$$

(41) $$N^{\prime }=\frac{Mg}{1+d^{\prime }/d^{\prime \prime }}\left(1-\frac{r}{d^{\prime }}\tan\alpha \cos\beta \right)$$

(42) $$N^{\prime \prime }=\frac{Mg}{1+d^{\prime \prime }/d^{\prime }}\left(1+\frac{r}{d^{\prime }}\tan\alpha \cos\beta \right)$$

From the other equations it results:

(43) $$\varepsilon =\frac{Mgr\sin\alpha \sin\beta -s_{r}{\left(\frac{Mg}{d^{\prime }+d^{\prime \prime }}\cos\alpha \right)}^{\lambda }\left[{\left(d^{\prime }+r\tan\alpha \cos\beta \right)}^{\lambda }+{\left(d^{\prime \prime }-r\tan\alpha \cos\beta \right)}^{\lambda }\right]}{J_{Cy}+Mr^{2}}$$

(44) $$T_{x}^{\prime }=-\frac{J_{Cy}\sin\alpha \sin\beta }{\left(1+\frac{J_{Cy}}{Mr^{2}}\right)\left(1+\frac{d^{\prime }}{d^{\prime \prime }}\right)}\cdot \frac{g}{r^{2}}+\frac{s_{r}\left(N^{\prime \lambda }+N^{\prime \prime \lambda }\right)}{\left(1+\frac{J_{Cy}}{Mr^{2}}\right)\left(1+\frac{d^{\prime }}{d^{\prime \prime }}\right)}$$

(45) $$T_{x}^{\prime \prime }=-\frac{J_{Cy}\sin\alpha \sin\beta }{\left(1+\frac{J_{Cy}}{Mr^{2}}\right)\left(1+\frac{d^{\prime \prime }}{d^{\prime }}\right)}\cdot \frac{g}{r^{2}}+\frac{s_{r}\left(N^{\prime \lambda }+N^{\prime \prime \lambda }\right)}{\left(1+\frac{J_{Cy}}{Mr^{2}}\right)\left(1+\frac{d^{\prime \prime }}{d^{\prime }}\right)}$$

The Equations (44) and (45) are necessary for verifying the realization of pure rolling conditions in the two points of contact:

(46) $$T_{x}^{\prime }<\mu N^{\prime }\;\mathrm{and}\;T_{x}^{\prime \prime }<\mu N^{\prime \prime }$$

where μ is the sliding friction coefficient between the ball and the inclined plane. The relation is rewritten under the form:

(47) $$\varepsilon_{a}=\frac{Mgr\sin\alpha \sin\beta -s_{r}{\left(\frac{Mgd^{\prime }}{d^{\prime }+d^{\prime \prime }}\cos\alpha \right)}^{\lambda }{\left(1+\frac{r}{d^{\prime }}\tan\alpha \cos\beta \right)}^{\lambda }-s_{r}{\left(\frac{Mgd^{\prime \prime }}{d^{\prime }+d^{\prime \prime }}\cos\alpha \right)}^{\lambda }{\left(1-\frac{r}{d^{\prime \prime }}\tan\alpha \cos\beta \right)}^{\lambda }}{J_{Cy}+Mr^{2}}$$

The index “a” refers to the case when the cylindrical collar is asymmetrically positioned $d^{\prime }\neq \;d^{\prime \prime }$. Equation (47) represents the theoretical conclusion of the present work. The eccentricity and the exponent λ will respond to the question: is the dependence between the rolling friction moment and the normal force a linear or nonlinear relation?

For the situation when the collar is mounted symmetrically, $d^{\prime }=d^{\prime \prime }$, Equation (47) takes a simpler form:

(48) $$\varepsilon_{s}=\frac{Mgr\sin\alpha \sin\beta -s_{r}{\left(\frac{Mg}{2}\cos\alpha \right)}^{\lambda }{\left(1+\frac{2r}{d}\tan\alpha \cos\beta \right)}^{\lambda }-s_{r}{\left(\frac{Mg}{2}\cos\alpha \right)}^{\lambda }{\left(1-\frac{2r}{d}\tan\alpha \cos\beta \right)}^{\lambda }}{J_{Cy}+Mr^{2}}$$

It can be observed that both expressions of angular accelerations take, for the case of linear dependency (similar to Coulomb law, namely for λ=1), the following simple form:

(49) $$\varepsilon =\frac{Mgr\cos\alpha \left(\tan\alpha \sin\beta -s_{r}/r\right)}{J_{Cy}+Mr^{2}}$$

Expression (49) does not depend on the position of the collar on the rod, that is, the eccentricity would not influence the angular acceleration. For λ≠1, the expressions of angular acceleration differ and the eccentricity influences the law of motion. These conclusions of the analytic study are the basis of the present work. 

### 2.2. Design Principles of the Test Rig

#### 2.2.1. Obtaining the Law of Motion 

A special problem to be solved is accurately finding the motion of the rotor, assumed to be constant when the rotor rolls without sliding on the inclined plane. For precise determining the law of motion of the rotor, a non-contact method was proposed, consisting in recording the motion of the rotor with a mobile camera. After that, the movie is analyzed using QuickTime Player software which splits into frames the record. Therefore, the time dependency of the angle of rotation of the rotor is established. The first attempts were made filming with a fixed camera, but it was noticed that the precision of establishing the position of a marker on the rotor decreases significantly when the distance rotor-camera increases. Therefore, the mobile camera was used in filming (Figure 5). The camera is placed downstream the rotor, on a trolley with wheels rolling down the inclined plane. When the motion of the rotor starts, the trolley with the camera is manually entrained. Due to the manual actuation of the trolley, the distance between the camera and the rotor is not constant and the variation of this distance depends on the efficacy of the human operator.

**Figure 6:**
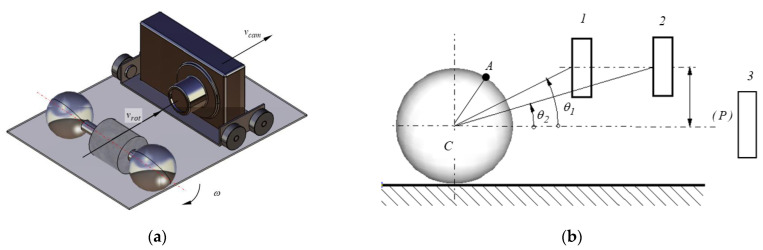
Principle of the moving camera: (**a**) schematics of moving rotor and mobile camera; (**b**) method for avoiding the parallax error.

A side schematic view of the test rig is presented in Figure 6 where *C* is the center of the rotor and the camera is presented in two distinct positions. The position of a marker attached to the rigid will be appreciated with respect to the plane by the axis of the rotor and the center of the camera lens. When the axis of the lens is eccentric with respect to the plane (*P*) passing through the axis of the rotor, parallel with the support plane, Figure 6, positioning the camera at different distances from the rotor will lead to a series of errors in the estimation of the rotation angle. To eliminate this drawback, the lens must be placed in the plane (*P*). Thus, the variation of the distance camera-rotor will not affect the position of the reference plane defined by the axis of the rotor and the center of the lens. 

**Figure 7:**
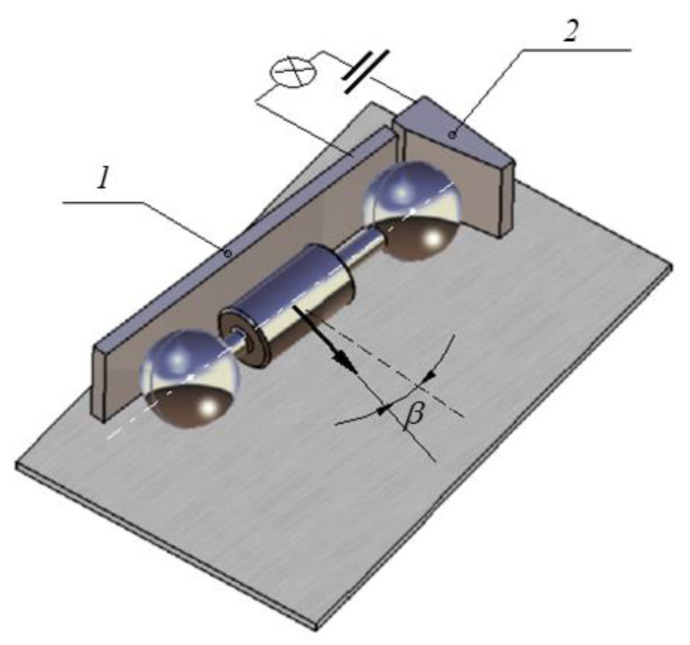
The launching position of the rotor: 1, 2 plates of the contact sensor.

Another important aspect concerns the precise identification of the instants when the motion of the rotor on the incline starts. The assumption that the rolling friction torque is constant during the period of motion allows for interpolating the time variation of the rotation angle with a parabola. For a better interpolation, the precise initial moment must be known. To this purpose, in the initial position, when the rotor is in the highest position of the inclined plane, one of the balls contacts the two small metallic plates, electrically insulated, and keeps closed an electrical circuit with a LED involved (Figure 7). When the rotor starts to move, the circuit is opened and the LED is off. 

#### 2.2.2. The Rolling Assembly

**Figure 8:**
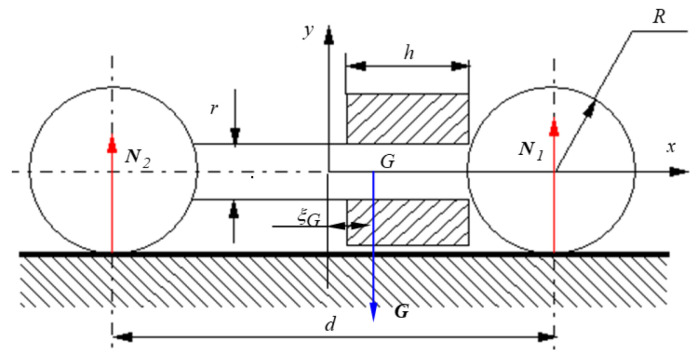
Eccentric rotor.

In designing the rotor, it is extremely important to accomplish the two situations mentioned previously referring to the normal forces $N_{1}$ and $N_{2}$, specifically when they are equal or different. In the case of dissimilar normal reactions, it is desirable for a greater difference in order to evidence as much as possible the effect of nonlinear dependency between the rolling friction torque and the normal force. The metallic collar is responsible for obtaining the difference between the two normal reactions, as represented in Figure 8. The bearing balls are made of steel of density $\rho =7800\;\mathrm{kg}/m^{3}$ and have the radius R=0.02 m, the mass $m_{b}=0.261\;\mathrm{kg}$ and the centric axial moment of inertia $J_{b}=3.746\times 10^{-6}\;\mathrm{kg}\cdot m^{2}$. The centers of the balls are maintained at a distance d=0.14 m by an aluminum rod of radius r=0.006 m and length (d−2·R)=0.1 m. The density of the aluminum is $\rho_{Al}=2700\;\mathrm{kg}/m^{3}$, so the rod has a mass $m_{r}=0.031\;\mathrm{kg}$ and a centric axial moment of inertia $J_{r}=5.479\times 10^{-7}\;\mathrm{kg}\cdot m^{2}$. The metallic collar has 1 mm smaller radius than the ball, so the contact between the rotor and the inclined plane is made with the two balls. The collar is made from steel, has a length denoted *h* and then the position of the center of mass of the rotor, with respect to the frontal plane of symmetry of the balls-rod assembly, $\xi_{G}$, is determined.

**Figure 9:**
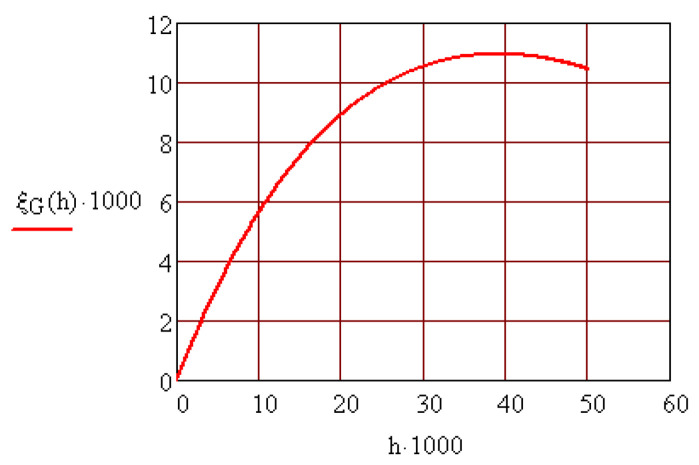
Optimizing the construction of the rotor from the dependency $\xi_{G}=\xi_{G}\left(h\right)$.

The dependency $\xi_{G}=\xi_{G}\left(h\right)$ is represented in Figure 9. It is observed that there is a value of the length *h* of the collar that ensures maximum eccentricity of the center of mass of the rotor.

With known eccentricity $\xi_{G}$ one can now determine the magnitudes of the normal reactions $\;N_{1}$ and $N_{2}$:

(50) $$N_{1}=\left(d/2+\xi_{G}\right)\left(2m_{b}+m_{r}+m_{c}\right)/d$$

(51) $$N_{2}=\left(d/2-\xi_{G}\right)\left(2m_{b}+m_{r}+m_{c}\right)/d$$

and their ratio, respectively:

(52) $$\frac{N_{1}}{N_{2}}=\frac{d/2+\xi_{G}}{d/2-\xi_{G}}.$$

**Figure 10:**
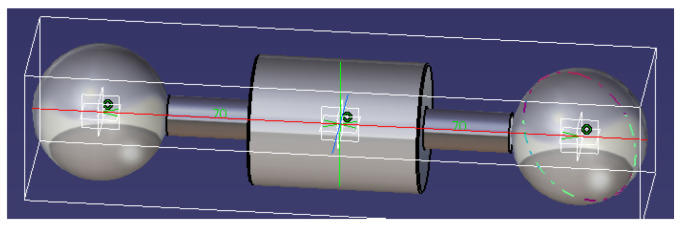
CAD model of the rotor.

In order to increase the values of the normal forces, the parameter h=0.05 m was adopted and then a mass of the collar $m_{C}=0.398\;\mathrm{kg}$ and an axial moment of inertia $J_{C}=7.904\times 10^{-5}\;\mathrm{kg}\cdot m^{2}$ resulted. With these values, the mass of the rotor was established analitically as M=0.954 kg and the axial moment of inertia $J_{Cy}=1.632\times 10^{-4}\;\mathrm{kg}\cdot m^{2}$. These results were validated by modeling the rotor using CATIA software as shown in Figure 10. The software provides the values for inertia characteristics: $M=0.958\;\mathrm{kg}\;\mathrm{and}\;J_{Cy}=1.644\times 10^{-4}\;\mathrm{kg}\cdot m^{2}$, confirming the analytical model.

## 3. Experimental Tests

### 3.1. Methodology

**Figure 11:**
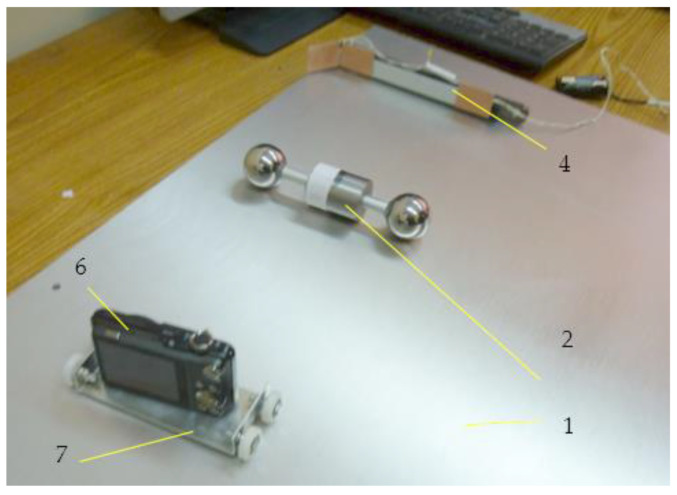
Experimental set-up.

**Figure 12:**
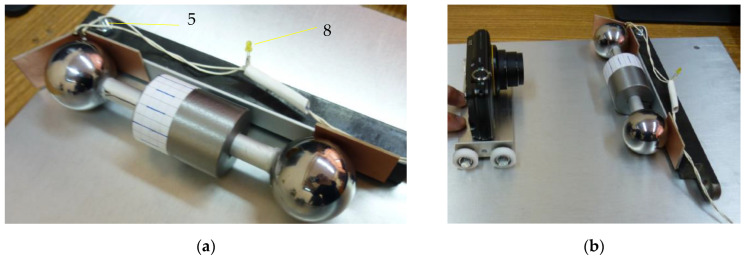
Details of the experimental set-up: (**a**) The rotor in initial launching position; (**b**) The launching instant (the LED is off) and the camera record the motion.

The experimental device constructed following the design principles mentioned above is presented in Figure 11 and Figure 12. It consists of a rectangular plate 1, the rotor 2 made of two identical bearing balls, the rod 3 used for tilting the plane, the prismatic body 4, the screw 5 that positions the prismatic body, the video camera 6, the trolley 7 that moves the camera and the LED 8 used as a sensor for the start of the displacement. The inclined plane 1 is a rectangular aluminum plate 12 mm thick, supported on one side. The inclination of the plate is obtained by supporting it on the opposite side on a steel rod 3, of 10 mm diameter. The tilting angle of the plane can be changed by moving the rod with respect to the supporting side, and the angle is precisely known. On the surface of the plane, on the supported side, a prismatic metallic body 4 is placed. The launching position of the rotor is when the spheres are in contact with this body. Thus, the rotor is launched from the desired position and orientation required for ensuring the same initial conditions. 

On the surface of the prismatic body, four electronic circuit boards are attached in order to diminish the effect of residual magnetic fields existing in the supporting body. An electric circuit plays the role of contact sensor: two plates were connected and the circuit is closed as long as the rotor is immobile. The circuit is aimed to identify the instant when the rotor starts to move. When the motion starts, the circuit is open and a LED from the circuit is off. 

The balls are connected by a rod with conical frontal surfaces, which ensure that when the ball is in contact with the cone, the center of the sphere is positioned on the axis of the collar and, therefore, the condition of a balanced rotor is satisfied. Before the balls are mounted, the adjustable collar with a radius smaller than the radius of the spheres is attached on the rod. 

An optical method was chosen for finding the law of motion. To this end, on the surface of the collar, a sheet of adhesive paper with equidistant marks was applied. A camera records the motion of the rotor at 120 frames/second and this film is used for determining the law of motion. The first attempts of filming were made with a fixed camera, but the images from a greater distance were unclear and the position was not precisely established. Then, the filming with a mobile camera was performed, the camera being placed on a trolley. An essential condition in the design of the trolley is to ensure the equality between the radius of the balls and the distance from the axis of the camera lens and the surface of the inclined plane. 

The trolley with the camera was brought in the vicinity of the rotor. The camera was started and the rotor was let free. After the rotor starts moving, the trolley was conducted, maintaining the relative position with respect to the rotor. A few exercise launchings were made and the angles α and β were adjusted to values that ensure displacement of the rotor as slow as possible. The slow displacement condition is essential because it is the necessary condition that assures that the rolling friction torque is comparable to the driving torque due to the tangential component of the weight. 

After recording the motion, the movie is analyzed using software and the time dependency of the angle of rotation of the rotor is established. As mentioned, the first attempts were made filming with a fixed camera, but it was noticed that the precision of establishing the position of a marker on the rotor decreases significantly when the distance rotor-camera increases. Therefore, the mobile camera was used in filming, Figure 12. The camera is placed downstream the rotor, on a trolley with wheels rolling over the inclined plane. When the motion of the rotor starts, the trolley with the camera is manually entrained. Due to the manual actuation of the trolley, the distance between the camera and the rotor is not constant and the variation of this distance depends on the efficacy of the human operator. By splitting the film into frames, one can precisely identify the instants when the index of the frame must be read, namely when the mark overlaps the axis of the rod. It is to be noted that the moment when the mark from the cylinder and the axis of the rod are superposed is independent of the position of the camera due to the construction of the trolley. The law of motion is deduced by identifying the instants corresponding to angles of rotation multiple of π/2.

### 3.2. Experimental Results

#### 3.2.1. Surface Quality

**Figure 13:**
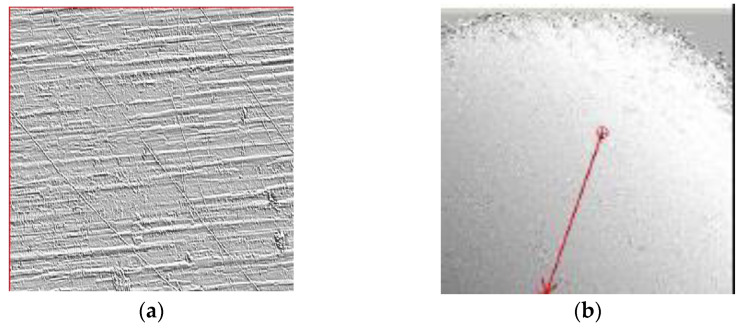
Results of the laser scan profilometry of the surfaces: (**a**) aluminum plate; (**b**) steel ball: the red arrow marks the direction and length of scanning.

**Figure 14:**
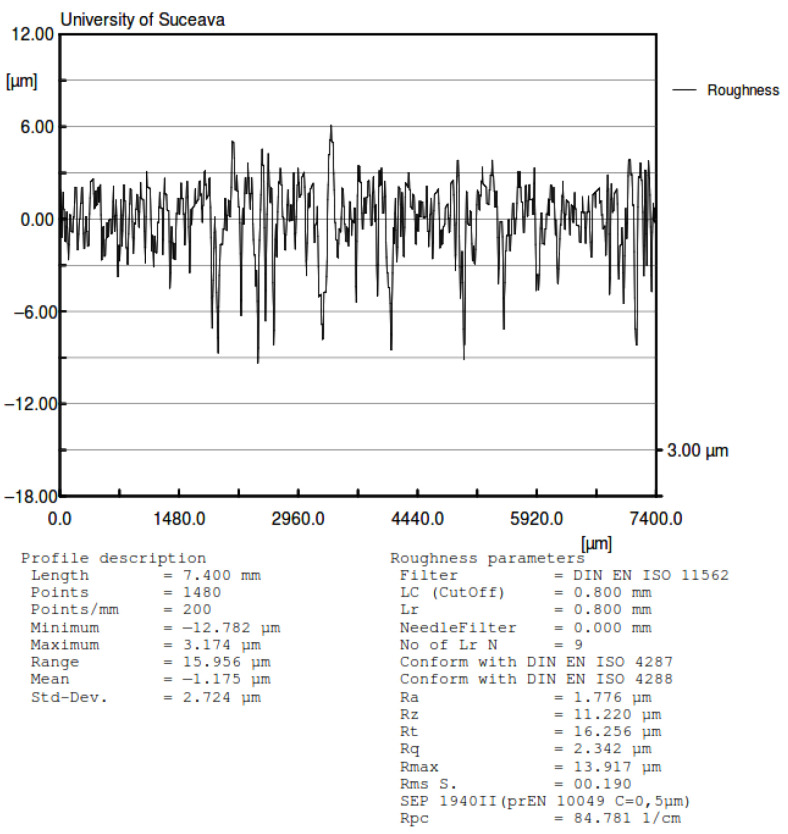
Profile of the aluminum plate with values of roughness.

**Figure 15:**
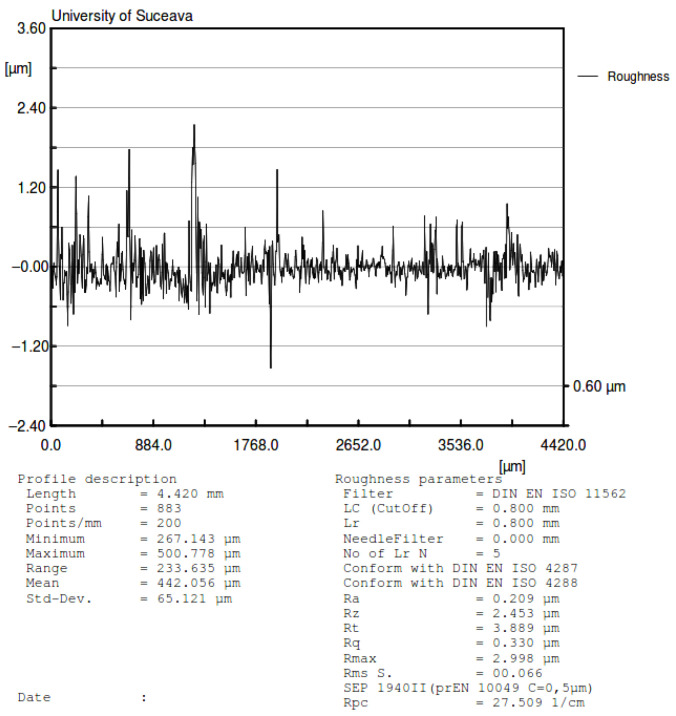
Profile of the steel ball with values of roughness.

**Figure 16:**
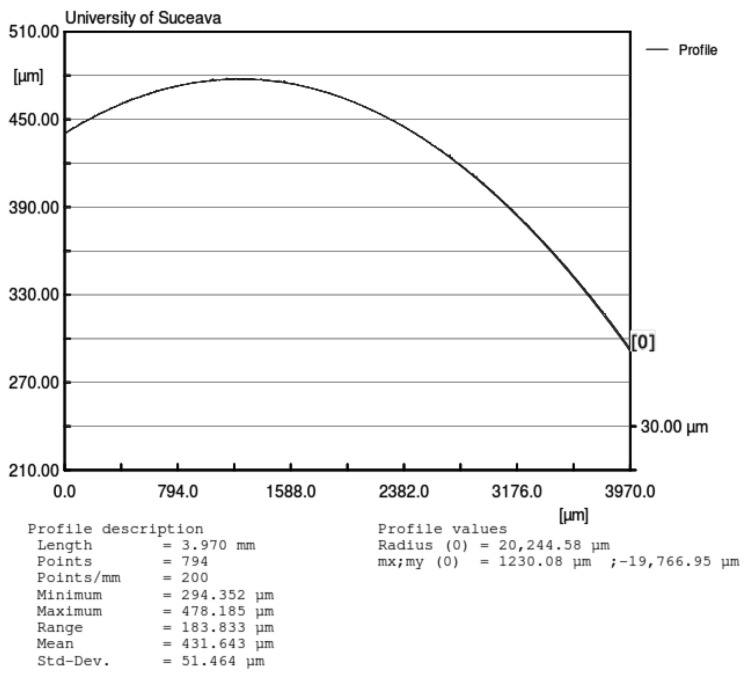
Experimental measurement of the radius of the ball.

Considering the fact that one of the reference papers, due to Cherepanov, that obtains a nonlinear dependency between the rolling friction torque and the normal force is based on the Hertzian contact theory, which assumes smooth surfaces of the contacting bodies, the experimental validation of this hypothesis was an objective for the present study. Notably, the parameters of the contact area from contact theory are not always experimentally validated [40,41]. To this end, a ball, identical to the one used for the device and a region from the aluminum plate were analyzed by laser scan profilometry, using the Nano Focus μScan Non-Contact Optical Surface Profilometer. The results of the laser scan profilometry of the surfaces of the two bodies are presented in Figure 13, Figure 14, Figure 15 and Figure 16. It should be mentioned that the constructed rotor was launched in rolling on the tested plate in order to see how the tips of the asperities are affected by the rolling rotor. In order to evaluate the quality of the contact surfaces, a specimen of the aluminum plate identical to the one used in tests was investigated with the aid of the laser profilometer. For the representation from Figure 13, a rectangular area having 8.063 mm × 11.309 mm was measured with 0.005 mm scanning resolution along both directions. A 2D profile was extracted and represented, as is shown in Figure 13. The profile direction was chosen in such a way that the wear trace obtained by contact between moving steel ball and aluminum plate to be perpendicular on the profile representation in order to highlight the deformations of the asperities. The following values of the 2D profile were obtained: *Ra* = 1.776 µm; *Rz* = 11.220 µm; *Rt* = 16.256 µm. The surface of a steel ball (identical with those used in tests) was measured in order to evaluate the surface roughness and geometrical deviations. It was investigated a rectangular area on the steel ball having 7.327 mm length along one direction and 7.580 mm length in the second direction. The resolution of the measurement is 0.05 mm along both directions. In Figure 13b, the red arrow shown is automatically represented by the laser profilometer software µScan-version 6.4; the arrow marks the direction and the length of the 2D profile represented in Figure 15. Measurements of the aluminum plate were made in different regions of the specimen and in similar conditions, but for representations of average values of the roughness parameters were chosen (Figure 14). Figure 14 was used to highlight the differences between the roughness parameters of the steel ball and aluminum plate. The results show that the steel ball has much smaller asperities. The surface of the steel ball can be assimilated as a smooth surface in contact with a rough surface (aluminum plate surface). 

The laser profilometer software is capable of evaluating only two parameters of the surface topography: Sa, Sq (from the Sa, Sq, Sp, Sv, Ssk, Sku, Spd) which describe the surface quality. In Table 1 there are presented the obtained values of the surface roughness parameters using µScan software.

The same profile from Figure 14 was used to determine the real ball dimension. The laser profilometer software has the capability to interpolate curves on the profile. In Figure 15, the interpolation (curved red line-circle) of the measured asperities is represented. The radius of the represented sector of the circle is the average profile of the ball.

Three measurements of the aluminum plate were made in different regions of the specimen and in similar conditions (5 µm scanning resolution). The average values of the roughness parameters were chosen for representations in Figure 13 and Figure 14. The obtained values for each measurement are presented in Table 2. Regarding the steel ball, three measurements were made on the surface in different regions, in similar conditions (50 µm scanning resolution) and the results are also presented in Table 2.

The Hertz theory [1] applied for the ball-plate contact for the case of a normal force N on the ball equal to the rotor weight (10 newtons), gives the maximum approach:

(53) $$\delta ={\left(\frac{9PN^{2}}{16R}\right)}^{1/3}=0.936\;\mu m$$

and the radius of the contact area is:

(54) $$a={\left(\frac{3}{4}NRP\right)}^{1/3}=136\;\mu m$$

From the values obtained, the normal approach is significantly smaller than the height of asperities from both contacting bodies, as given by profilometry (Figure 14 and Figure 15). Since the Hertzian theory assumes smooth contacting surfaces, and in our case, the asperities are greater than the normal approach, it can be concluded that it is a sensible matter if the contact theory can be applied in this case [41]. 

**Figure 17:**
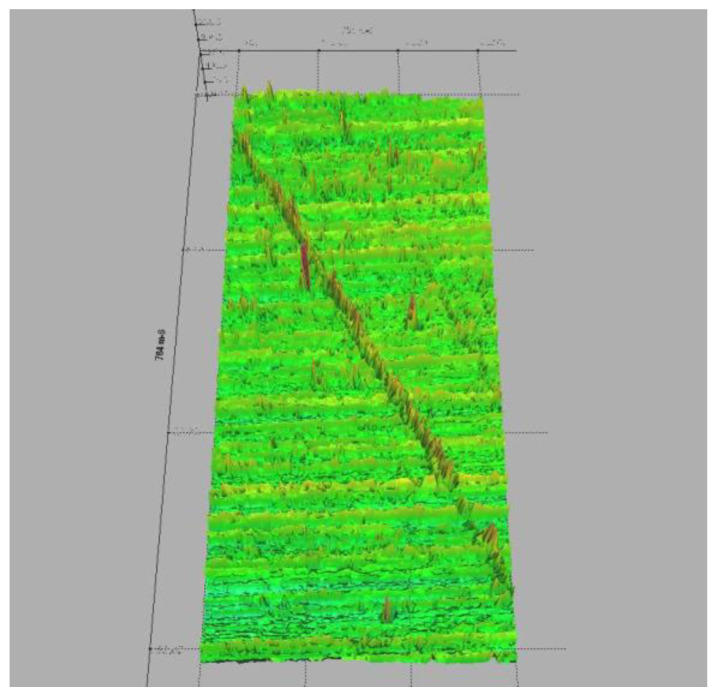
The plastic deformations of the plate along the rolling trajectory obtained by the scanner reflectivity method.

An increased normal force should give greater approaches, but it also conducts to larger contact area and plastic deformations. From Figure 17, the reflectivity method evidences the plastic deformations even for the normal forces given by the weight of the rotor. As a conclusion, from Figure 13, Figure 14, Figure 15, Figure 16 and Figure 17, it can be observed that the hypothesis of smooth contacting bodies is far from being satisfied and, therefore, the value of the exponent *λ* cannot be considered known from the contact theory in the calculus of the rolling friction torque. 

Another conclusion emerging from the analysis of the profiles is that there are shape deviations of the balls, the experimental radius differing from the theoretical one (Figure 16). Thus, the radius variation means a change of the distance from the center of mass of the rotor to the plate surface and will operate as a rotor eccentricity. This phenomenon may present important effects, especially for small inclination angles of the plane [42]. 

#### 3.2.2. The Law of Motion

The linear or nonlinear form of the dependency between the rolling friction torque and the normal force is tested by determining the angular displacement of the rotor. Two situations of launchings were considered: the cylinder placed centric and the cylinder placed for maximum eccentricity. To adjust the position of the cylinder, the balls of the rotor were each one placed on a weighing scale and by measuring the weights, the eccentricity is controlled, as in Figure 5.

**Figure 18:**
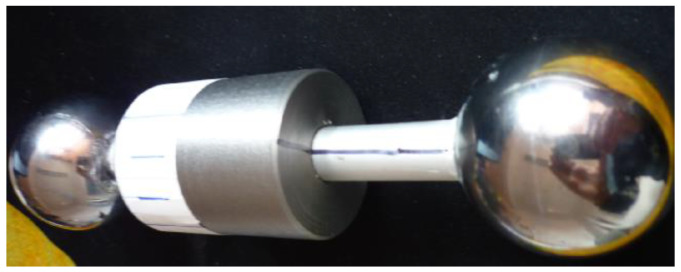
Maintaining the position of the center of mass of the rotor with respect to the axis of symmetry of the rotor.

The ratio between the masses displayed by the two weighing scales is the ratio between the normal forces and, based on the Equations (50) and (51), permits finding the values of the position of the center of mass $\xi_{G}$ and, $d^{\prime }$, $d^{\prime \prime }$ implicitly. Assuming that it is possible that, due to manufacturing and construction errors and to materials non-homogeneities, a deviation of the center of mass from the axis of the rotor may exist, two marks were traced: a generator line on the rod and a radial line on the front of the collar, Figure 18. These marks should always intersect in order to keep the position of the center of mass.

**Figure 19:**
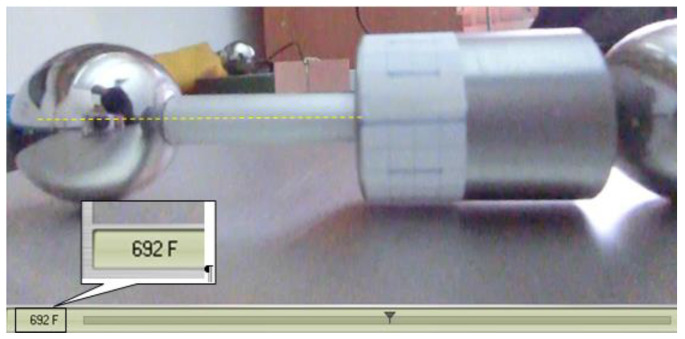
Identification of movie frame index for the rolling rotor.

The movies obtained, split into frames, are analyzed as described above and the moments corresponding to rotations multiple of π/2 of the rotor are identified (Figure 19). 

Three launchings were made for each of the two positions of the collar—symmetrical rotor and asymmetrical rotor and the indices of the frames are presented in Table 3. The absolute time corresponding to a frame is calculated with the relation:

(55) $$t_{k}=\left(f_{k}-f_{1}\right)/120$$

where $f_{k}$ is a value from row No. *k* from Table 3. 

**Figure 20:**
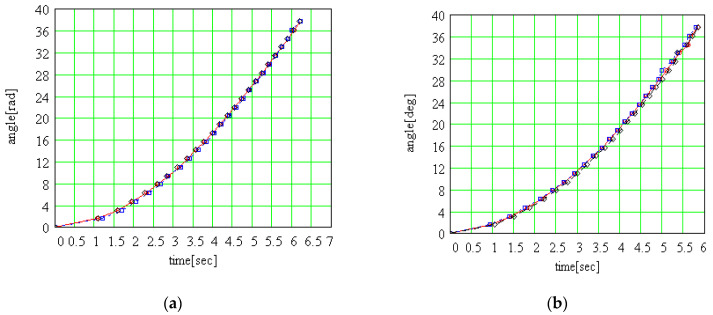
Experimental rolling angle: (**a**) symmetrical rotor; (**b**) asymmetrical rotor.

**Figure 21:**
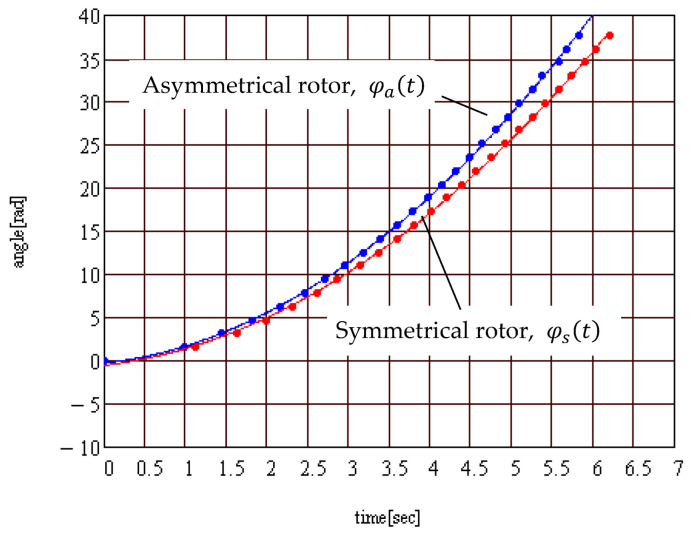
Time dependency of the rolling angle for the symmetrical and asymmetrical rotor.

The plots of the angle of rotation as a function of time are presented in Figure 20. They show very good repeatability of results. For the two different positions of the collar, the points lay on different curves, confirming that the moment of rolling friction was greater for the eccentric collar case. The mean values for symmetrical and asymmetrical rotor were used to compare two cases, Figure 21. The theoretical results show that the angular acceleration of the rotor is constant and the angle of rotation has a parabolic variation. Considering that the rotor starts from the still situation, it is normal that the function of interpolation contains only the quadratic term. But, by interpolating the data from Figure 21 with a general form parabola, one can notice that both the free term and the coefficient of the term of first degree take non-zero values:

(56) $$\varphi_{s}\left(t\right)=0.821t^{2\;}+1.134t-0.527;\varphi_{a}\left(t\right)=0.951t^{2\;}+1.0254t-0.249$$

This observation raises the doubt that the experimental results were affected by errors. As error sources, one can remind:

- The eccentric position of the center of mass of the rotor, due to materials inhomogeneities, shape errors, and relative position errors;
- The identification of the moment corresponding to a specified rotation of the rotor;
- The identification of the initial moment of motion;
- Possible errors of planarity of the aluminum plate.

From the plots in Figure 21, it is obvious that there is a difference between the two cases for symmetrical and asymmetrical rotor, and this fact proves the nonlinear dependency between the rolling friction torque and the normal force. 

### 3.3. Discussion

When dry friction is considered between two contacting bodies, the Amonton–Coulomb law characterizes the friction force is linearly dependent on the normal force and independent on the dimensions of the contact area. The first models for the rolling friction torque use the analogy with the dry friction force and assume linear dependency between the rolling friction torque and normal force. This hypothesis is convenient since it conducts to simpler dynamic models of the systems where rolling friction is present. 

In order to explain the presence of rolling friction, unlike for the sliding friction case, the hypothesis of a rigid body must be abandoned. Thus, when the normal force acts, the two bodies deform and a region of contact of small dimensions occurs. The normal reaction, which is the sum of elementary forces on the contact surface, in a general case will no longer intersect the instantaneous axis of rotation of each body. When the normal reaction is eccentric with respect to the axis of rotation, it will generate a moment that opposes the relative rotation between the two bodies: the rolling friction torque.

For the viscoelastic bodies, the model of the normal force assumes a damping component which makes plausible the nonlinear dependency between the rolling friction torque and the normal force. Additionally, in practical applications with rolling bodies made of viscoelastic materials, the contact deformations are significant, and the nonlinear effects can be evidenced by traditional investigation methods. 

For elastic materials, there are different aspects. The dissipative component of the reaction forces is lacking and the only explanation for the presence of rolling friction remains the eccentric position of the normal reaction with respect to the axis of rotation. Considering that for usual elastic contacting materials such as steel, glass etc. The radius of the contact area is tens of microns in size, it is expected that the eccentricity of the reaction with respect to the axis of rotation should be of a tenth of microns in size. Therefore, a conclusion is that for elastic materials, the rolling friction torque has very small values, and special methods and instruments are required for experimental study. 

There are two main techniques for the dynamic study of rolling friction systems with oscillatory motions and with non-oscillatory motions. The oscillatory motion methods are based on the effect of angular amplitude diminishing of the specially designed pendula. The non-oscillatory motion methods are founded on the braking effect produced by the rolling friction upon a body rolling on an inclined plane. 

The angular acceleration for a special rotor, in pure rolling, that moves along an imposed direction—different from the line of the highest slope, on an inclined plane is found. This motion is correspondent to the motion on an incline of a much smaller angle. The advantage is that the angle of the equivalent plane can be precisely determined analytically from the values of the actual inclined. This small angle of the inclined is required in order to make perceptible the effect of rolling friction. 

The special principle of the rotor permits the change only for the position of the center of mass, but maintains unmodified the mass and the inertia matrix. The centric and eccentric cases were studied and the optimum eccentric position was established. For a certain eccentricity, the equation of motion of the rotor on the inclined plane was deduced under the hypothesis that the rolling friction moment is proportional to the normal reaction raised at a power λ. The equation of motion highlights that for a linear dependency, for  λ = 1, the value of the angular acceleration does not depend on the position of the collar on the rod. For λ≠1, for different eccentricities, the accelerations change. 

A special rotor was designed and experiments were made in order to obtain the law of motion. Due to errors of machining, material inhomogeneities, and other causes, it is most probable that an eccentricity of the center of mass of the rotor with respect to the axis of rotation exists, and it may present significant effects for small angles of inclination of the plane. For this reason, on the connecting rod a generatrix was traced and on the frontal face of the cylinder a mark was made, and the overlap of these ensures the repeatability of the experiments. 

For the experimental finding of the law of motion, a non-contact method was used by recording the motion of the rotor with a high-speed camera and analyzing the fames of the movie. The device constructed for the moving camera eliminates the parallax errors and ensures a good image for the marks on the rotor. 

Another necessary analysis concerns the quality of the contacting surfaces. To this end, the surfaces of the balls and of the plane were scanned by laser profilometry, and it was observed that the smooth surfaces hypothesis, required by Hertzian theory, is far from being satisfied and, thus, the value for λ = 4/3 from contact mechanics theory is not applicable. 

## 4. Conclusions

- The paper highlights experimentally the nonlinear dependency between the rolling friction torque and the normal reaction from a Hertzian point contact.
- The rolling friction torque occurring between elastic materials has very small values and requires specific devices for experimental analysis.
- The theoretical contribution of the paper consists in finding the angular acceleration for a special rotor in pure rolling on an inclined of small angle, necessary to make observable the effect of rolling friction.
- The centric and eccentric case for the special rotor is considered and the optimum eccentric position is established. The equation of motion of the rotor is deduced under the hypothesis that the rolling friction moment is proportional to the normal reaction raised at a power *λ*. The equation of motion specifies that for linear dependency (*λ* = 1), the value of the angular acceleration does not depend on eccentricity. For λ≠1, for different eccentricities, the acceleration varies.
- The special rotor was designed and constructed and the experimental law of motion was obtained from the analysis of the frames of recorded motion.

For future research, the next goals are proposed:

- In order to obtain better experimental results, the errors must be diminished. The radial eccentricity can be reduced by using two rotors more precisely machined—a symmetrical one and an asymmetrical one—but with the same inertial characteristics and after performing a balancing process. The measurement of the errors of planarity of the plate and a theoretical study concerning the manner these errors influence the law of motion, assumed parabolical, are also considered necessary.
- The use of software for image analysis can offer a more precise identification of the moments corresponding to a stipulated rotation.
- The use of an oscilloscope can establish more accurately the initial moment of the motion.

## Figures and Tables

**Table 1:** Surface roughness parameters of the contact surfaces.

No.	Part	Sa	Sq
1	Aluminum plate	1.255 µm	5.340 µm
2		1.186 µm	1.541 µm
3		1.415 µm	5.820 µm
1	Steel ball	1.203 µm	4.818 µm
2		0.999 µm	2.447 µm
3		1.415 µm	5.820 µm

**Table 2:** Two-dimensional roughness parameters of the contact surfaces.

No.	Part	Ra	Rz	Rt	Rq
1	Aluminum plate	1.776 µm	11.220 µm	16.256 µm	2.342 µm
2		1.263 µm	9.277 µm	11.337 µm	1.629 µm
3		2.165 µm	13.566 µm	13.566 µm	2.639 µm
1	Steel ball	0.209 µm	2.453 µm	3.889 µm	0.330 µm
2		0.230 µm	1.109 µm	2.553 µm	0.329 µm
3		0.192 µm	2.137 µm	3.948 µm	0.290 µm

**Table 3:** Experimental data for finding the rolling angle of the rotor.

	Asymmetrical Rotor Frame Index			Symmetrical Rotor Frame Index		
No.	Test 1 as	Test 2 as	Test 3 as	Test 1 s	Test 2 s	Test 3 s
1	2071	231	45	965	1	1226
2	2200	375	175	1082	113	1351
3	2263	433	234	1137	168	1406
4	2307	477	278	1183	213	1451
5	2348	516	317	1223	256	1492
6	2384	552	354	1257	291	1526
7	2417	574	386	1288	323	1558
8	2447	614	416	1318	352	1587
9	2475	641	445	1344	379	1614
10	2500	667	472	1371	404	1639
11	2526	692	498	1395	429	1663
12	2550	715	523	1417	451	1686
13	2573	737	546	1439	473	1707
14	2596	760	569	1461	494	1729
15	2618	782	591	1481	515	1749
16	2639	803	612	1501	535	1769
17	2660	823	633	1521	554	1789
18	2681	844	654	1540	573	1808
19	2701	864	673	1559	591	1826
20	2721	884	693	1577	600	1844
21	2740	903	713	1595	627	1864
22	2760	922	732	1613	645	1869
23	2780	941	751	1638	664	1896
24	2799	951	770	1647	678	1912
25	2818	978	788	1667	695	1928
